# Therapeutic plasma exchange in heart transplantation: role of coagulation assessment with thromboelastometry

**DOI:** 10.1186/s40981-016-0058-1

**Published:** 2016-10-19

**Authors:** Andrew Crabbe, John S. McNeil, Seema P. Deshpande, Zachary Kon, Si M. Pham, Kenichi A. Tanaka

**Affiliations:** 1 Department of Anesthesiology, University of Maryland, 22 South Greene Street, Suite S8D12, Baltimore, MD 21201 USA; 2 Department of Anesthesiology, University of Virginia, Charlottesville, VA USA; 3 Department of Surgery, Division of Cardiothoracic Surgery, University of Maryland, Baltimore, MD USA

**Keywords:** Coagulation, Plasma exchange, Fresh frozen plasma, Coagulation factor deficiency, Heart transplantation

## Abstract

Therapeutic plasma exchange (TPE) is a potentially life-saving procedure which effectively removes donor-specific human leukocyte antigen (HLA) antibodies from the bloodstream, allowing critically ill heart transplant recipients to receive a donor organ with less wait time, and reducing the risk of acute organ rejection. The bulk of coagulation factors is initially removed from the blood during TPE using albumin and is later replaced with allogeneic plasma. Coagulopathy may develop during TPE and then can persist due to intraoperative blood loss and hemodilution during surgery and cardiopulmonary bypass. We hereby describe the utility of rotational thromboelastometry to assess rapid coagulation changes during TPE and subsequent heart transplant (HT) surgery.

## Background

Therapeutic plasma exchange (TPE) is a procedure that is used to remove potentially harmful antibodies and toxins from a patient. It is indicated for many disease conditions including autoimmune anemia, autoimmune vasculitis, hemolytic uremic syndrome, and multiple sclerosis [1]. TPE has been used in perioperative cardiac surgical patients with antibodies relating to heparin-induced thrombocytopenia [2], ABO incompatibility [3], and donor-specific human leukocyte antigen (HLA) [4]. The latter antibodies (ABO and HLA) are pertinent to organ donor matching, and transplant recipients may suffer from long waits for organs due to incompatible antibodies. Perioperative removal of these antibodies via TPE is feasible and often life-saving for critically ill organ recipients.

Albumin (5 %) is useful as an exchange fluid for serial TPEs because of potential risks associated with a large amount of fresh frozen plasma (hereinafter referred to as plasma) [5]. In particular, allergic reactions can occur in up to 40–65 % of patients undergoing multiple TPEs, although the severity of symptoms may vary [5–7]. It is thus routine at our institution to perform two plasma volume exchanges using 5 % albumin, followed by another TPE with plasma. The loss of coagulation factors during TPE is expected [8], but there is no strong evidence to support the superiority of plasma-only TPE over albumin/plasma combination for non-surgical TPE indications [5, 9]. Perioperative TPEs using albumin/plasma raise concerns about coagulation status because a single TPE with plasma may be insufficient to fully restore hemostatic factor levels before the use of cardiopulmonary bypass (CPB). Rotational thromboelastometry (TEM Innovations, Munich) was thus used to assess rapid coagulation changes during repeat TPEs in this case of a patient with donor-specific HLA antibodies who underwent a heart transplantation. The aim of this report is to address the clinical implications of TPE on coagulation and hemodynamic management in the transplant recipient.

## Case presentation

A 43-year-old female, 66 kg, with non-ischemic cardiomyopathy (NICM) presented for HT. She was diagnosed with NICM 15 years ago, presumably related to pregnancy and/or systemic lupus erythematous (SLE). Her past medical history included SLE with associated nephritis and vasculitis, insulin-dependent diabetes mellitus, previous pulmonary embolism, and previous stroke with residual visual field defects.

The patient had been stable on a biventricular assist device (BiVAD; HeartWare, Framingham, MA, USA) for 2 years while no HLA-matched donor was found despite a 1b (moderate urgency) status. She was recently hospitalized for worsening right heart function and was requiring increased inotropic support. Given the decline in her clinical condition, the transplant team decided to use therapeutic plasma exchange (TPE), anti-lymphocyte antibody treatment, and intravenous immunoglobulin treatment in the perioperative period in order to accept a cross-match positive donor heart [4, 10].

**Fig. 1:**
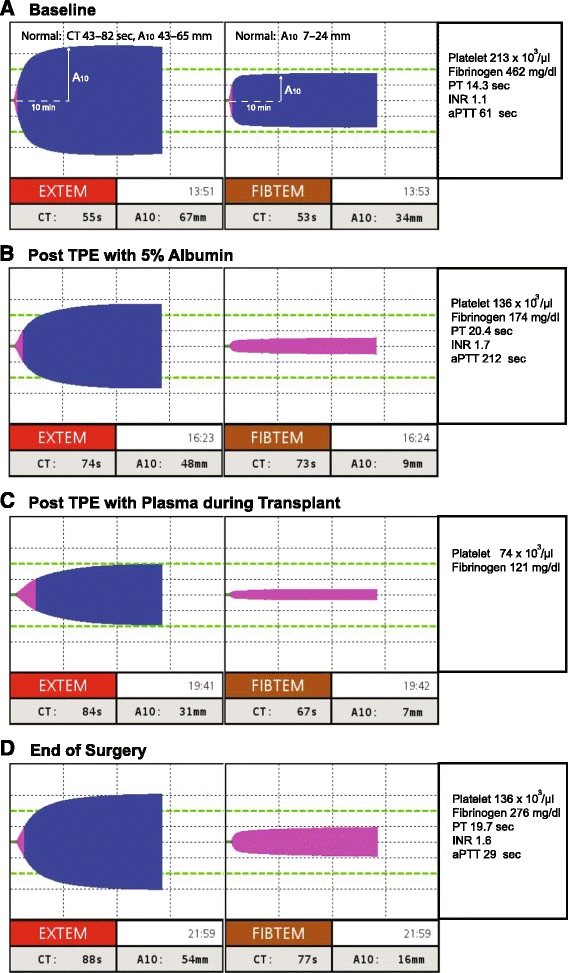
**a** Key parameters of EXTEM-CT: clotting time (s) [normal, 43–82 s], EXTEM-A_10_: 10 min amplitude (mm) [normal, 43–65 mm], and FIBTEM-A_10_ [normal, 9–24 mm]. **b**–**d** EXTEM and FIBTEM changes during and after therapeutic plasma exchange (TPE). Baseline fibrinogen was in supra-normal range (**b**), but it extensively decreased after TPE with albumin (**c**) and even after plasma replacement during surgery. Fibrinogen was restored only after the transfusion of cryoprecipitate (**d**)

After an uneventful induction of anesthesia and line placement, a large dual lumen catheter was placed in the femoral vein and connected to the plasmapheresis device (Prismaflex® System, GAMBRO, Colorado). She underwent TPEs consisting of three plasma volume exchanges; the first two exchanges used 5 % albumin (3300 ml each) to remove the HLA antibodies. The third plasma exchange utilized 11 units of FFP to restore coagulation factors for surgery. Throughout the procedure, coagulation status was assessed using tissue factor-activated EXTEM and FIBTEM on rotational thromboelastometry (Fig. 1). Baseline (pre-TPE) EXTEM and FIBTEM parameters were within normal range except for a high FIBTEM-A_10_. After TPE with albumin, EXTEM-CT increased from 55 to 74 s. FIBTEM-A_10_ decreased from 34 to 9 mm (−64.0 %) (Fig. 1a, b). TPE continued with FFP (~35 ml/kg) during the explantation of the BiVAD device, and FIBTEM-A_10_ was 7 mm at the conclusion of this third round of TPE (Fig. 1c). Measured plasma fibrinogen levels, which were reported approximately 25–45 min after FIBTEM-A_10_ results were available, confirmed a net loss of fibrinogen (Fig. 1a, c).

The donor heart was implanted over 192 min of CPB with a cross-clamp time of 93 min and a donor ischemic time of 256 min. After transplant and reperfusion, the patient was initially weaned from CPB with inotropic support (epinephrine 0.06 μg/kg/min and norepinephrine 0.03 μg/kg/min). Diffuse microvascular bleeding was attributed to thrombocytopenia (EXTEM-A_10_, 31 mm) and low fibrinogen (FIBTEM-A_10_, 7 mm) (Fig. 1c). One pool of five single donor platelets and 20 units (four pools) of cryoprecipitate were transfused, and improved hemostasis was observed in the surgical field in conjunction with normalized EXTEM and FIBTEM A_10_ values (Fig. 1d).

In the post-CPB period, systolic dysfunction persisted on increasing doses of epinephrine and norepinephrine (up to 0.1 μg/kg/min) despite adding vasopressin (0.06 units/min) and milrinone (0.375 μg/kg/min). Levothyroxine (T_4_; 20 μg/kg bolus, followed by 5 μg/kg/h infusion) was added to treat “stunned” myocardium due to the long ischemic time as well as the potential for “euthyroid sick syndrome” exacerbated by TPE [11, 12].

The transplant heart’s systolic function improved with the T_4_ infusion, and further mechanical support (e.g., veno-arterial extracorporeal membrane oxygenation) was avoided. The patient remained stable in the intensive care unit (ICU), and the T_4_ infusion was weaned off along with other inotropes on postoperative day (POD) 2. In the ICU, four more sessions of TPE were performed to sustain the eradication of donor-specific antibodies; one plasma volume exchange with FFP on POD 1 and 2, and one plasma volume exchanged with 5 % albumin on POD 3 and 5 (plasma fibrinogen levels were above 150 mg/dL after TPEs). The patient was extubated on POD 5 and discharged from the hospital on POD 14.

### Discussion

Rotational thromboelastometry is a practical method to assess rapid coagulation changes during surgery and CPB, and in this case report, it was used successfully during multiple TPEs for heart transplantation (HT). [13]. Tissue factor-activated tests, EXTEM and FIBTEM (Table 1), are most useful in the acute setting because extrinsic coagulation factors and fibrinogen, respectively, are particularly prone to hemodilution and blood (plasma) losses [14, 15]. Clotting time (CT) of EXTEM reflects prothrombin time (PT) sensitive coagulation factor levels [15], whereas 10 min amplitude (A_10_ clot firmness) reflects platelet count and fibrinogen level [16]. FIBTEM better reflects plasma fibrinogen levels than EXTEM because its clot formation is fibrin specific due to the inclusion of cytochalasin D, a platelet inhibitor, in the reagent [16, 17].

**Table 1:** Types of assays on rotational thromboelastometry

Test	TF	Contact	Heparin effect	Detectable condition(s)	Hemostatic interventions
EXTEM	+		−^a^	Extrinsic pathway, PLT count, fibrinolysis	Plasma, PCC, or PLTs
FIBTEM	+		−^a^	Fibrinogen level^b^	Cryo or fibrinogen conc.
APTEM	+		−^a^	Systemic fibrinolysis^c^	Antifibrinolytics
INTEM		+	+	Intrinsic pathway, PLT count, fibrinolysis	Plasma, protamine, or PLTs
HEPTEM		+	−^a^	Heparin effect	Same as INTEM

*ACT* activated clotting time, *Cryo* cryoprecipitate, *Fibrinogen conc.* fibrinogen concentrate, *PCC* prothrombin complex concentrate, *PLT* platelet, *TF* tissue factor activator, *Contact* contact activator (ellagic acid)

^a^Heparin is neutralized by the reagent at concentrations up to 4–6 U/ml on EXTEM/FIBTEM/APTEM and 8 U/ml on HEPTEM

^b^Confirmatory test for hypofibrinogenemia; low clot firmness (amplitude) on other ROTEM® tests may indicate either thrombocytopenia or hypofibrinogenemia

^c^Confirmatory test for fibrinolysis; fibrinolysis may be shown on all the other ROTEM® tests

TPE is an important intervention for a variety of diseases that require the removal of undesirable toxins or antibodies. TPE potentially expands the donor pool for heart and lung transplants which otherwise can be limited by recipient antibodies to donor HLA. One to one and a half plasma volume runs of TPE generally remove 63–78 % of alloantibodies (mostly IgG class), and three TPEs can reduce these antibodies by 90 % [18]. In conjunction with immunosuppressive agents, TPE can significantly reduce the risk of both acute and chronic allograft organ rejection [4]. Reduced FIBTEM values correlate with the substantial removal of large plasma proteins (IgG, 150 kDa, and fibrinogen, 340 kDa) at the end of albumin TPE [8]. Loss of fibrinogen and other coagulation proteins during albumin TPE increase the risk of bleeding, particularly after CPB, and thus allogeneic plasma is generally recommended for intraoperative TPEs [2]. However, full recovery of fibrinogen is unlikely even if plasma-only TPE is used [5, 9], and CPB-induced hemodilution further decreases plasma fibrinogen [17]. Post-CPB fibrinogen levels below 200 mg/dL (FIBTEM-A_10_ < 10 mm) are associated with an increased risk of bleeding and higher transfusion requirement [19, 20], and the restoration of fibrinogen to >250 mg/dL (FIBTEM-A_10_ > 15-18 mm) appears to reduce such risks [21]. We initially calculated the required amount (g) of fibrinogen using the published formula below based on the FIBTEM-A_10_ value [21–23]:

$$ \begin{array}{l}\mathrm{Fibrinogen}\ \left(\mathrm{g}\right)\kern0.37em  = \left[\mathrm{target}\ \mathrm{FIBTEM}\hbox{-} {\mathrm{A}}_{10}\left(\mathrm{mm}\right)\ \hbox{--}\ \mathrm{current}\ \mathrm{FIBTEM}\hbox{-} {\mathrm{A}}_{10}\left(\mathrm{mm}\right)\right] \times \mathrm{weight}\ \left(\mathrm{kg}\right)\div 140\\ {} = \left(15\ \mathrm{mm}\ \hbox{--}\ 7\ \mathrm{mm}\right) \times 66\ \mathrm{kg}\div 140 = 3.77\ \mathrm{g}\end{array} $$

In the USA, clinical use of fibrinogen concentrate is limited to hereditary afibrinogenemia and hypofibrinogenemia [24], and thus cryoprecipitate remains the mainstay therapy for perioperative fibrinogen replacement. The required amount (units) of cryoprecipitate can be estimated by multiplying the fibrinogen dose by five [25]; 3.77 × 5 = 18.9 units.

After transfusing 20 units of cryoprecipitate, FIBTEM-A_10_ and fibrinogen were 16 mm and 276 mg/dL, respectively.

Our case demonstrates the practical use of EXTEM and FIBTEM in monitoring perioperative changes in fibrinogen levels and the therapeutic effects of cryoprecipitate (Fig. 1c, d) during TPE. It is also important to note that antibiotics (ceftazidime, tobramycin, etc.) and immunosuppressants (basiliximab) can also be eliminated by TPE [26, 27].

In addition, TPE is known to affect biologically active hormones, including free thyroxine (T_4_) and triiodothyronine (T_3_) [12]. Critically ill patients have limited reserves of thyroid hormones [11], and there is some evidence to suggest that there is better preservation and function of the donor heart using T_3_ or T_4_ infusion [28]. Therefore, T_4_ infusion may be potentially useful for the management of stunned donor heart.

## Conclusions

In conclusion, the use of TPE expands the donor organ pool to patients who otherwise would have an either a low probability of or a long wait before receiving a donor organ. In these patients, thromboelastometry throughout the perioperative period can allow for a fast detection of coagulopathy and targeted component therapy [25, 29].
